# Heterogeneous Antimicrobial Susceptibility Characteristics in *Pseudomonas aeruginosa* Isolates from Cystic Fibrosis Patients

**DOI:** 10.1128/mSphere.00615-17

**Published:** 2018-03-14

**Authors:** Xuan Qin, Chuan Zhou, Danielle M. Zerr, Amanda Adler, Amin Addetia, Shuhua Yuan, Alexander L. Greninger

**Affiliations:** a Department of Laboratory Medicine, University of Washington, Seattle, Washington, USA; b Department of Pediatrics, University of Washington, Seattle, Washington, USA; c Seattle Children’s Hospital, Seattle, Washington, USA; U.S. Centers for Disease Control and Prevention

**Keywords:** *Pseudomonas aeruginosa*, cystic fibrosis, heterogeneous, heteroresistance, isogenic, syntrophic

## Abstract

Clinical isolates of *Pseudomonas aeruginosa* from patients with cystic fibrosis (CF) are known to differ from those associated with non-CF hosts by colony morphology, drug susceptibility patterns, and genomic hypermutability. *Pseudomonas aeruginosa* isolates from CF patients have long been recognized for their overall reduced rate of antimicrobial susceptibility, but their intraclonal MIC heterogeneity has long been overlooked. Using two distinct cohorts of clinical strains (*n* = 224 from 56 CF patients, *n* = 130 from 68 non-CF patients) isolated in 2013, we demonstrated profound Etest MIC heterogeneity in CF *P. aeruginosa* isolates in comparison to non-CF *P. aeruginosa* isolates. On the basis of whole-genome sequencing of 19 CF *P. aeruginosa* isolates from 9 patients with heterogeneous MICs, the core genome phylogenetic tree confirmed the within-patient CF *P. aeruginosa* clonal lineage along with considerable coding sequence variability. No extrachromosomal DNA elements or previously characterized antibiotic resistance mutations could account for the wide divergence in antimicrobial MICs between *P. aeruginosa* coisolates, though many heterogeneous mutations in efflux and porin genes and their regulators were present. A unique OprD sequence was conserved among the majority of isolates of CF *P. aeruginosa* analyzed, suggesting a pseudomonal response to selective pressure that is common to the isolates. Genomic sequence data also suggested that CF pseudomonal hypermutability was not entirely due to mutations in *mutL*, *mutS*, and *uvr*. We conclude that the net effect of hundreds of adaptive mutations, both shared between clonally related isolate pairs and unshared, accounts for their highly heterogeneous MIC variances. We hypothesize that this heterogeneity is indicative of the pseudomonal syntrophic-like lifestyle under conditions of being “locked” inside a host focal airway environment for prolonged periods.

**IMPORTANCE** Patients with cystic fibrosis endure “chronic focal infections” with a variety of microorganisms. One microorganism, *Pseudomonas aeruginosa*, adapts to the host and develops resistance to a wide range of antimicrobials. Interestingly, as the infection progresses, multiple isogenic strains of *P. aeruginosa* emerge and coexist within the airways of these patients. Despite a common parental origin, the multiple strains of *P. aeruginosa* develop vastly different susceptibility patterns to actively used antimicrobial agents—a phenomenon we define as “heterogeneous MICs.” By sequencing pairs of *P. aeruginosa* isolates displaying heterogeneous MICs, we observed widespread isogenic gene lesions in drug transporters, DNA mismatch repair machinery, and many other structural or cellular functions. Coupled with the heterogeneous MICs, these genetic lesions demonstrated a symbiotic response to host selection and suggested evolution of a multicellular syntrophic bacterial lifestyle. Current laboratory standard interpretive criteria do not address the emergence of heterogeneous growth and susceptibilities *in vitro* with treatment implications.

## Abstract (precis)

Patients with cystic fibrosis endure “chronic focal infections” with a variety of microorganisms. One microorganism, *Pseudomonas aeruginosa*, adapts to the host and develops resistance to a wide range of antimicrobials. Interestingly, as the infection progresses, multiple isogenic strains of *P. aeruginosa* emerge and coexist within the airways of these patients. Despite a common parental origin, the multiple strains of *P. aeruginosa* develop vastly different susceptibility patterns to actively used antimicrobial agents—a phenomenon we define as “heterogeneous MICs.” By sequencing pairs of *P. aeruginosa* isolates displaying heterogeneous MICs, we observed widespread isogenic gene lesions in drug transporters, DNA mismatch repair mechanisms, and many other structural or cellular functions. Coupled with the heterogeneous MICs, these genetic lesions demonstrated a symbiotic response to host selection and suggested evolution of a multicellular syntrophic bacterial lifestyle. Current laboratory standard interpretive criteria do not address the emergence of heterogeneous growth and susceptibilities *in vitro* with treatment implications.

## INTRODUCTION

In the airway environment of patients with cystic fibrosis (CF), *Pseudomonas aeruginosa* and other common bacterial cohabitants undergo host airway niche adaption and virulence attenuation (1, 2). When the organism first enters the CF airway, it is under enormous host selective pressure. This pressure leads to a number of well-described phenotypic and genotypic adaptive features such as an overall slow growth rate, diverse colony variants, heterogeneous antimicrobial susceptibility patterns, adoption of a biofilm or multicellular lifestyle, and the emergence of hypermutators, which result from nonsynonymous changes or loss of function mutations in *mutS*, *mutL*, and/or *uvr* (3–6).

Hospital antibiograms are routinely constructed by clinical microbiology laboratories annually (7). The format of hospital antibiograms is standardized by reporting “percent susceptible” using interpretive criteria recommended by professional advisory organizations such as the Clinical and Laboratory Standards Institute (CLSI) (8). Additionally, for clinical laboratories constructing institutional antibiograms, CLSI recommends inclusion of only the first isolate of a given species per patient per reporting period irrespective of body site, antibiotic susceptibility, or other phenotypic characteristics (8). This selection has unintended implications with respect to our understanding of a subset of clinical cultures. For example, unlike the pseudomonal isolates from non-CF patients, pseudomonal colony variants from CF patients producing highly variable and constantly changing susceptibilities are frequently encountered but not accounted for (9–11). Thus, the biological characteristics associated with this subset of organisms have been overlooked.

In the case of CF *P. aeruginosa* infection, research attention has been paid not only to high-level antimicrobial resistance but also to the phenomena of antimicrobial hypersusceptibilities, heteroresistance, and heterogeneous susceptibilities between isogenic coisolates (9, 12–15). For example, high-level resistance to beta-lactams, including carbapenems, in CF *P. aeruginosa* has long been linked to acquisition of corresponding hydrolytic enzymes, inactivation or alteration of the drug transporter porin D, and many adaptive mutations in regulatory determinants such as *mexR*, *nalC*, and *nalD*. Mutations in these regulatory elements were previously demonstrated to contribute to the hyperexpression of *mexAB-oprM*, encoding a prominent drug-proton antiporter or a resistance-nodulation-cell division (RND)-type multidrug efflux pump (16–18). However, our understanding of the mechanism(s) associated with a paradoxical CF *P. aeruginosa* multiagent hypersusceptibility phenomenon *in vitro* remains limited (9, 13). In our previous study, we reported that *P. aeruginosa* isolates with heterogeneous sulfamethoxazole-trimethoprim (SMX-TMP) MICs from the same CF patient were isogenic by pulsed-field gel electrophoresis (PFGE) (9). Moreover, we found mixed *mexAB-oprM* alleles among clonally coexisting *P. aeruginosa* isogenic isolates that were associated with heterogeneous SMX-TMP MICs (19–21). As previously proposed (9, 14, 22), the CF airway-specific bacterial colony variants may have assumed an interdependent multicellular mode of replication and resistance. Once a founder clone diverges to show diverse colony morphotypes, auxotrophism, and adaptive *lasR* inactivation within a *P. aeruginosa* lineage, therapy based on *in vitro* susceptibilities is a poor predictor of clinical outcome and the organism is nearly impossible to eradicate (15, 23–28).

**FIG 1:**
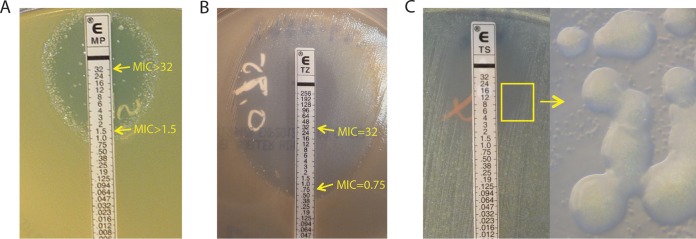
*P. aeruginosa* “strain-specific” heteroresistance. (A) Inner colonies growing above the MIC level where the predominant growth was inhibited at a meropenem concentration of 1.5 μg/ml. E MP, meropenem Etest. (B) Two subpopulations of the same *P. aeruginosa* isolate showing two distinct ceftazidime MICs, 0.75 and 32 μg/ml. E TZ, ceftazidime Etest. (C) Heterogeneous growth of two subpopulations (big and small) of *P. aeruginosa* as seen under a microscope with total magnification of ×30. E TS, sulfamethoxazole-trimethoprime Etest.

Heteroresistance is generally described as a state represented by subpopulations of bacteria that exhibit a heterogeneous range of susceptibilities to a particular antibiotic in an otherwise clonal population (Fig. 1A and B) (14). The underlying concept of “subpopulations of bacteria” suggested differences within a bacterial clonal population that could not be further separated *in vitro* by selective subculture passages. In this study, we chose to use the term “heterogeneous MICs” to describe highly variable (>4-fold difference) *in vitro* MIC values generated by 2 or more *P. aeruginosa* isolates from the same culture with respect to at least one drug tested in common.

This investigation aimed to describe the heterogeneous *in vitro* MIC patterns found in CF *P. aeruginosa* isolates by inclusion of all colony variants from all cultures within the 7-month study period. Both within- and between-patient *P. aeruginosa* antimicrobial MIC variances were computed for comparisons between two cohorts of *P. aeruginosa* isolates (non-CF and CF). The mechanism of pseudomonal heterogeneous MICs and the proposed pseudomonal isogenic syntrophic growth and resistance were further explored by whole-genome sequencing of selected patient-specific *P. aeruginosa* isolate pairs.

## RESULTS

### Specimen characteristics of the two cohorts of *P. aeruginosa* isolates.

A total of 354 clinical *P. aeruginosa* isolates were included in this analysis: 224 CF *P. aeruginosa* isolates from 100 cultures obtained from 56 patients and 130 non-CF *P. aeruginosa* isolates from 99 cultures obtained from 68 patients (Table 1). The CF *P. aeruginosa* isolates were generally from sputum samples, throat swabs, and bronchoalveolar lavage fluid specimens. The non-CF *P. aeruginosa* isolates came from a variety of specimen types (tracheal aspirates [*n* = 41], bronchoalveolar lavage fluid [*n* = 3], urine [*n* = 24], wound [*n* = 8], ear canal [*n* = 7], blood [*n* = 5], cerebral spinal fluid [*n* = 3], sinus [*n* = 1], and stool [hematology-oncology patients] [*n* = 7]).

**TABLE 1:** Culture and isolate characteristics classified by CF and non-CF cohorts

*P. aeruginosa* cohort category	No. of patients	No. of cultures	No. of isolates	Avg no. (range) of isolates per culture	No. (%) of cultures with ≥2 isolates	No. (%) of cultures that had isolates with discordant susceptibilities^a^	Avg no. (range) of agents involved in discordant susceptibilities per culture^a^
CF	56	100	224	2.24 (1–5)	64 (64.00)	41/64 (64.06)	1.73 (0–10 in 64 cultures)
Non-CF	68	99	130	1.31 (1–3)	28 (28.28)	6/28 (21.43)	0.39 (0–3 in 28 cultures)

a The data for discordant susceptibilities include only those representing changes in MIC interpretations between “S” and “R.” Data corresponding to changes in MIC interpretations between “I” and “S” or between “I” and “R” are not included.

### Examples of heteroresistance and their frequencies in each cohort.

In contrast to the stable wild-type *P. aeruginosa* homogeneous growth, which is sustained through *in vitro* passages, heterogeneous growth and resistance patterns are frequently found and considered to be “mixed” in clinical laboratories (Fig. 1). Heterogeneous growth properties of CF *P. aeruginosa* can often be determined by the naked eye but sometimes can be recognized only under a microscope (Fig. 1C). In the most extreme example, this property of *P. aeruginosa* is defined as heteroresistance, as further subculturing of the isolate results in the same heterogeneous growth and resistance patterns.

The first example of heteroresistance can be common to both CF and non-CF *P. aeruginosa* isolates where “inner colonies” were growing above the meropenem MIC level of 1.5 μg/ml, the level at which the predominant population of the clone was inhibited (Fig. 1A). The second example of two subpopulations with an antimicrobial MIC at either 0.75 μg/ml or 32 μg/ml, respectively, was also found in CF *P. aeruginosa* isolates (Fig. 1B). In this study, for isolates generating MIC patterns similar to those shown in Fig. 1A and B, MICs of >32 μg/ml for meropenem (Fig. 1A) and 32 μg/ml for ceftazidime (Fig. 1B) were recorded, respectively, and their heteroresistance patterns were noted as part of the susceptibility record. During the study period, patterns of heteroresistance to at least one antimicrobial agent tested were found in 16 (7%) CF *P. aeruginosa* isolates and 4 (3%) non-CF *P. aeruginosa* isolates in our clinical microbiology laboratory. As the extreme heteroresistance phenotype is less frequent than that of the extensively heterogeneous MICs seen with CF *P. aeruginosa* isolates, we undertook a systematic analysis of differences in the levels of inter- and intrapatient *P. aeruginosa* antimicrobial MIC heterogeneity between two cohorts of CF and non-CF *P. aeruginosa* isolates.

### CF *P. aeruginosa* isolates show higher numbers of colony variants by culture and more-pronounced between-patient and within-patient MIC heterogeneity.

In order to examine between-patient, within-patient, and within-culture phenotypic heterogeneity of *P. aeruginosa* isolates, we specifically included multiple isolates from the same patient (whether from the CF or non-CF cohort). The term “coisolates” was used to describe ≥2 colony variant *P. aeruginosa* isolates from the same culture. The average number of *P. aeruginosa* coisolates per culture in the CF cohort was twice as high as in the non-CF cohort (Table 1). Overall, 64% of cultures from the CF cohort had ≥2 *P. aeruginosa* isolates compared to only 28% of cultures from the non-CF cohort (Table 1). Examining antibiotic susceptibilities, a higher proportion of coisolates from the CF cohort had discordant susceptibilities with respect to at least one antimicrobial agent than among those from the non-CF cohort (64% versus 21%). Further, the average number of agents with discordant susceptibilities was also higher among the *P. aeruginosa* coisolates from the CF cohort than among those from the non-CF cohort (1.73 versus 0.39) (Table 1).

**FIG 2:**
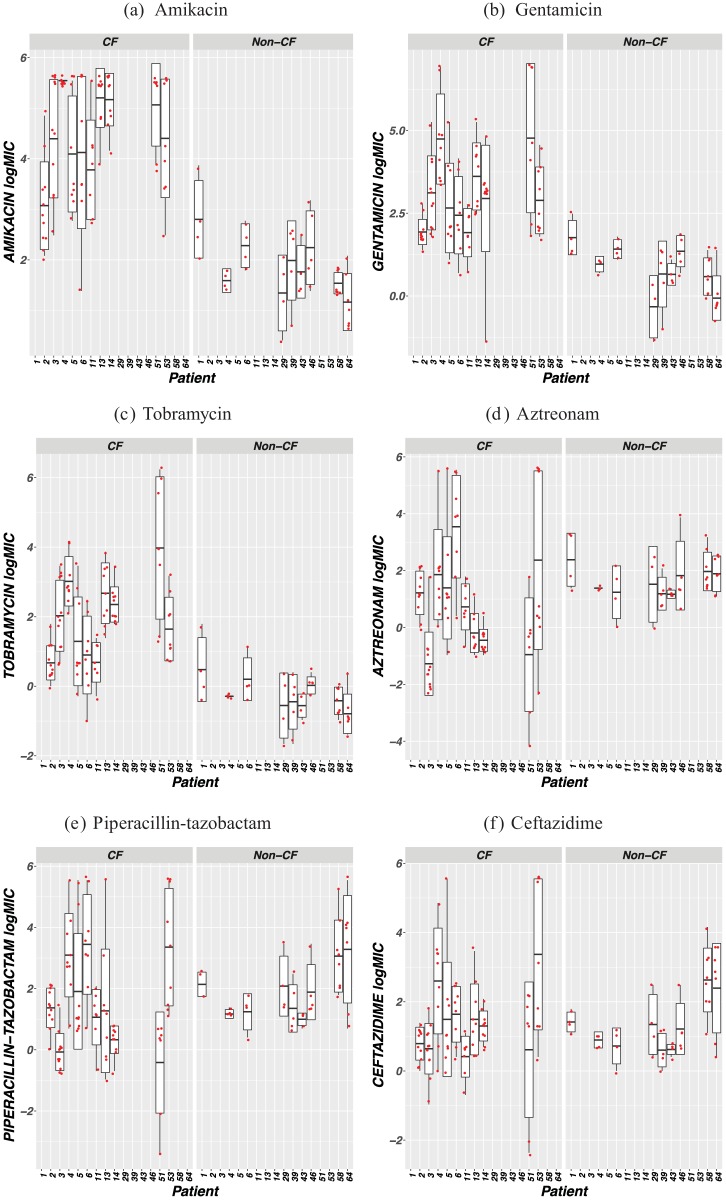

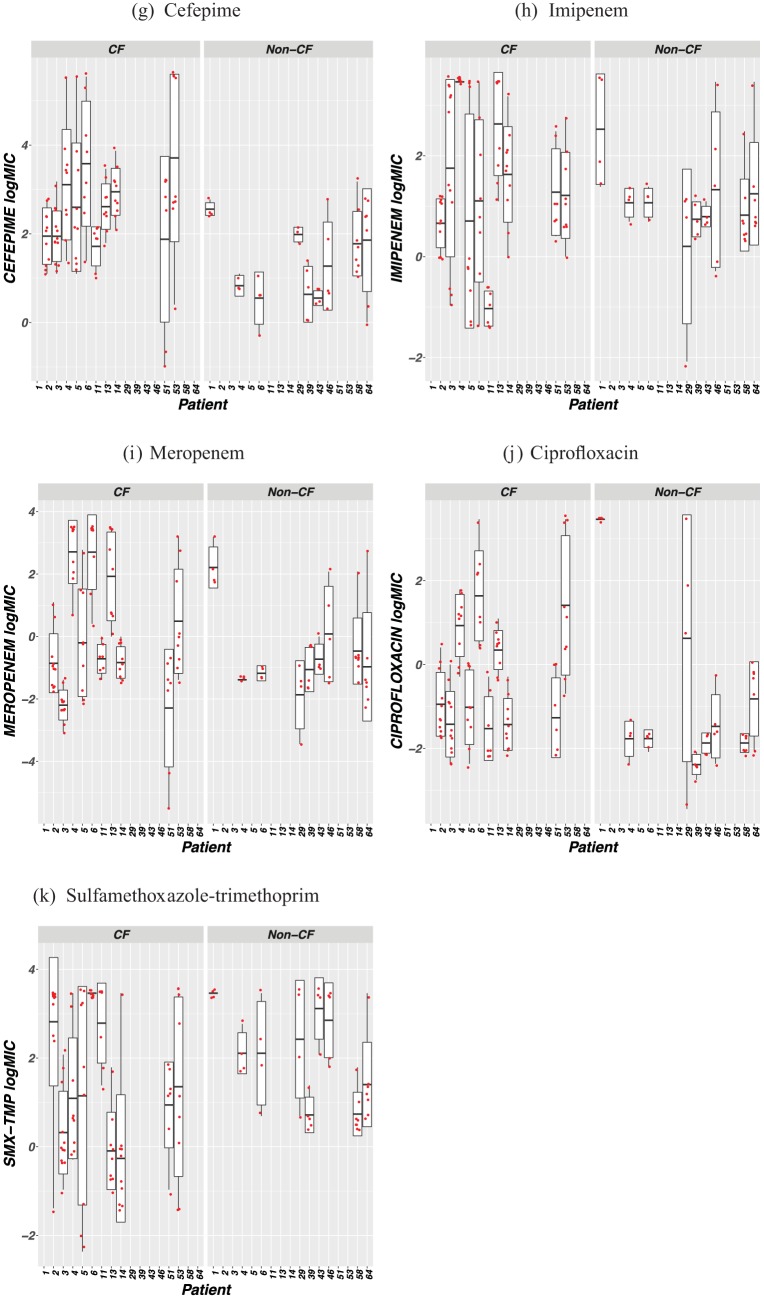
Antimicrobial logMIC distributions for *P. aeruginosa* isolates from selected patients in both CF and non-CF cohorts who contributed >4 isolates within the study period. The dots indicate the logMICs from patient-specific isolates, the vertical lines represent the range of logMICs, the box represents the lower (25%) quartile and upper (75%) quartile, and the horizontal line represents the median.

In order to collect exact MIC values, the wide-range Etest method was chosen for this study and logarithmic MIC values (logMICs) were calculated to allow quantitative comparisons between antimicrobial concentration ranges that would be otherwise difficult to accurately compare in the linear range (e.g., 0.002 to 32 µg/ml for meropenem versus 0.064 to 1,024 µg/ml for tobramycin). Comparisons of within-patient antimicrobial susceptibilities between cohorts are shown in Fig. 2 for patients who contributed more than four isolates within the study period. The within-patient logMIC heterogeneities appeared more pronounced in the CF cohort than in the non-CF cohort against all examined agents except for imipenem, meropenem, and ciprofloxacin (CIP) (Fig. 2).

Comparing total levels of variance of antimicrobial logMICs between *P. aeruginosa* isolates from the CF cohort and those from the non-CF cohort, significantly more heterogeneity was observed for amikacin, aztreonam, cefepime, piperacillin-tazobactam, and SMX-TMP in the isolates from the CF cohort (Table 2). The same comparisons also suggested a trend toward heterogeneity in the CF cohort for ceftazidime and imipenem, but the difference was not statistically significant.

**TABLE 2:**
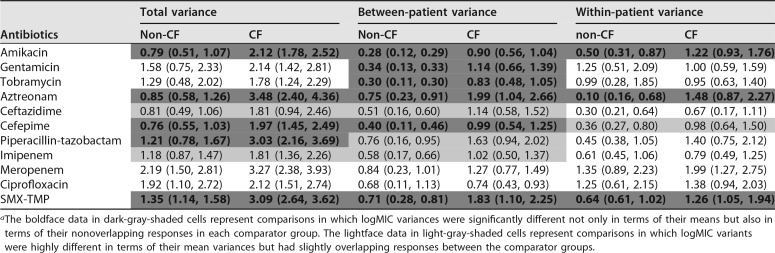
Within- and between-patient variances as well as total variances in logMICs for *P. aeruginosa* isolates associated with cultures from the two cohorts of patients, namely, the CF patients and non-CF patients^a^

a The boldface data in dark-gray-shaded cells represent comparisons in which logMIC variances were significantly different not only in terms of their means but also in terms of their nonoverlapping responses in each comparator group. The lightface data in light-gray-shaded cells represent comparisons in which logMIC variants were highly different in terms of their mean variances but had slightly overlapping responses between the comparator groups.

Comparing between-patient variances of antimicrobial logMICs between *P. aeruginosa* isolates from the CF cohort and those from the non-CF cohort, significantly more heterogeneity was observed for amikacin, gentamicin, tobramycin, aztreonam, cefepime, and SMX-TMP in the isolates from the CF cohort (Table 2). The same comparisons also suggested a trend toward heterogeneity in the CF cohort for ceftazidime, piperacillin-tazobactam, and imipenem, but the difference was not statistically significant.

Comparing within-patient variances of antimicrobial logMICs between *P. aeruginosa* isolates from the CF cohort and those from the non-CF cohort, significantly more heterogeneity was observed for amikacin, aztreonam, and SMX-TMP in isolates from the CF cohort (Table 2). The same comparisons also suggested a trend toward heterogeneity in the CF cohort for cefepime, but the difference was not statistically significant.

### Whole-genome sequencing of 9 sets of CF *P. aeruginosa* isolates revealed a within-patient clonal lineage in the setting of heterogeneous MICs *in vitro*.

**FIG 3:**
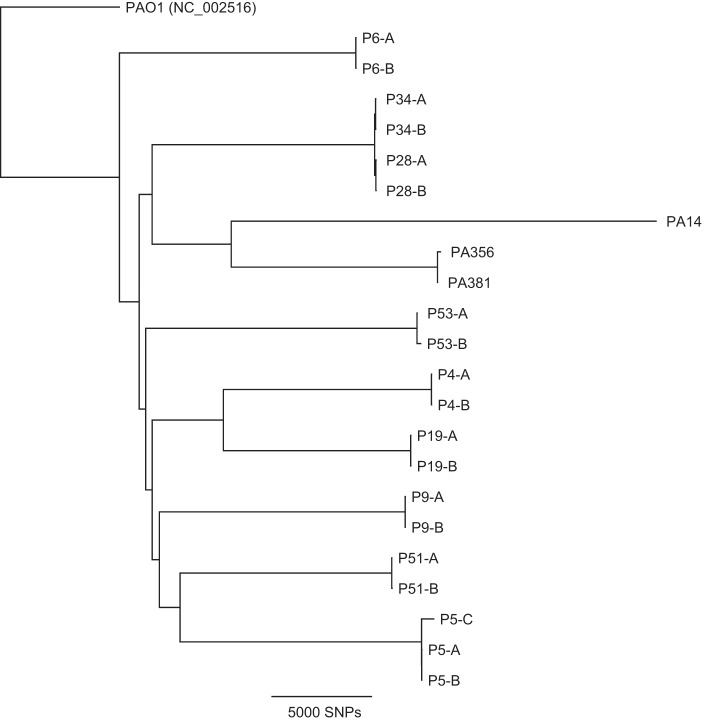
Core genome tree data confirming close intrahost relatedness of the 19 CF *P. aeruginosa* isolates. A core genome single nucleotide polymorphism (SNP) tree was created based on read mapping to the PAO1 reference genome. Additional* P. aeruginosa* reference genomes PA14 (wild type, non-CF), PA356 (CF), and PA381 (CF) are included in the tree. Each patient is represented by a designation consisting of a capital “P” followed by a number, and different isolates from the same patient are labeled with the letters A, B, and C.

To examine the molecular basis and clonal relatedness of *P. aeruginosa* isolates producing heterogeneous MICs, 19 CF *P. aeruginosa* isolates belonging to 9 patients (8 pairs of *P. aeruginosa* isolates from 8 patients and 1 set of 3 *P. aeruginosa* isolates from 1 patient) were selected for whole-genome sequencing. *P. aeruginosa* isolates were named based on the study patient number using a capital “P” followed by an isolate designation of A, B, or C (Table 3). All patient isolates selected for genome sequencing were either from the same cultures (patients 4, 6, 9, 19, 34, 51, and 53) or from different cultures from the same patient within the 7-month study period (patients 5 and 28) (Table 3). Genomes of both PAO1 and UCBPP-PA14 (PA14) were included as wild-type *P. aeruginosa* references, as both strains originated from non-CF sources (29, 30). Two genomes corresponding to CF *P. aeruginosa* strains *356 and *381 belonging to clonal type DK40 and representing isolates collected from two different patients whose data were reported in an earlier European study (10) and which clustered closer to PA14 than other isolates were also included for analysis (Fig. 3). In order to assist the annotation of variants selected in the CF host, sequence variants against the PAO1 reference genome that were also found in PA14 were treated as “neutral” changes for study strains, with the assumption that they would not result in major MIC discrepancies or in other CF *P. aeruginosa* phenotypic characteristics (Table 4 and 5; see also Table S1 to S4 in the supplemental material).

10.1128/mSphere.00615-17.1 TABLE S1  Polymorphic changes detected in gene determinants of RND and porin. Note that the gray-shaded rows represent the mutations that were shared with PA14 or present in PA14 only. Download TABLE S1, XLSX file, 0.04 MB. Copyright © 2018 Qin et al. 2018 Qin et al. This content is distributed under the terms of the Creative Commons Attribution 4.0 International license.

10.1128/mSphere.00615-17.2 TABLE S2  Summary of CDS polymorphic changes in chromosomal antibiotic resistance genes *ampC*, *ampD*, *ampD*, *ampG*, *dacB*, *bla*_OXA-50_, *aph(3′)-IIb*, and *cat*. Note that the gray-shaded rows represent the mutations that were shared with PA14 or present in PA14 only. Download TABLE S2, XLSX file, 0.02 MB. Copyright © 2018 Qin et al. 2018 Qin et al. This content is distributed under the terms of the Creative Commons Attribution 4.0 International license.

10.1128/mSphere.00615-17.3 TABLE S3  Polymorphic changes in genes governing DNA mismatch repair, nucleic acid synthesis, DNA or RNA modification and integrity, and DNA recombination functions in *P. aeruginosa* isolates. Note that the gray-shaded rows represent the mutations that were shared with PA14 or present in PA14 only. Download TABLE S3, XLSX file, 0.02 MB. Copyright © 2018 Qin et al. 2018 Qin et al. This content is distributed under the terms of the Creative Commons Attribution 4.0 International license.

10.1128/mSphere.00615-17.4 TABLE S4  Annotated CDSs with significant mutations, such as deletions, insertions, or frameshift or nonsense/stop codon truncations, among CF *P. aeruginosa* isolates. Note that those CDSs encoding hypothetical proteins and polymorphic changes that resulted in amino acid nonsynonymous substitutions were not included; the gray-shaded rows represent the mutations that were shared with PA14 or present in PA14 only. Download TABLE S4, XLSX file, 0.1 MB. Copyright © 2018 Qin et al. 2018 Qin et al. This content is distributed under the terms of the Creative Commons Attribution 4.0 International license.

**TABLE 3:**
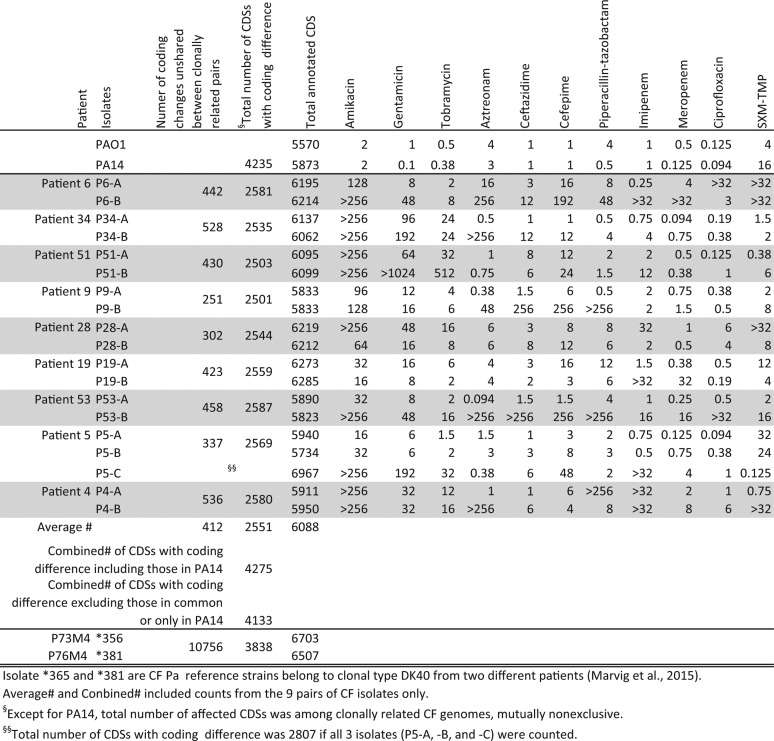
Summary of *in vitro* antimicrobial MICs and overall genomic polymorphisms of the 19 CF *P. aeruginosa* isolates and control strains that were subjected to whole-genome sequencing and comparison

**TABLE 4:**
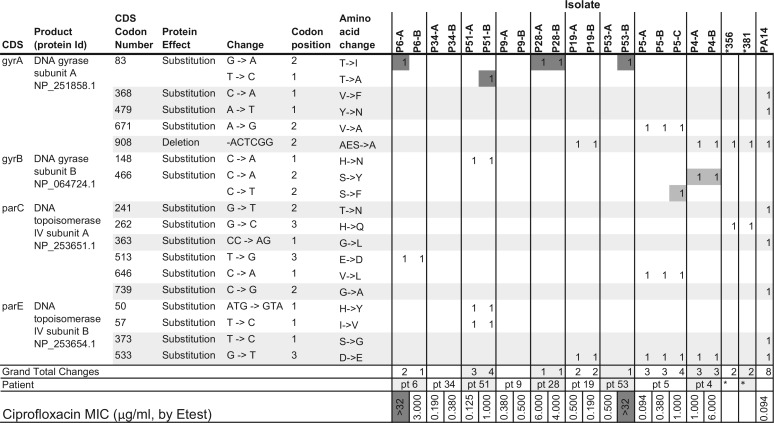
Polymorphic QRDR mutations detected in the 19 CF *P. aeruginosa* isolates with heterogeneous ciprofloxacin MICs, with the reference genomes of *356, *381, and PA14 included in the comparison^*a*^

a The well-known QRDR mutations and lesser-known mutations are represented in darker-gray and lighter-gray cells, respectively, while data in the lightest-gray rows represent polymorphisms that are shared with PA14. Id, identifier; pt, patient.

**TABLE 5:**
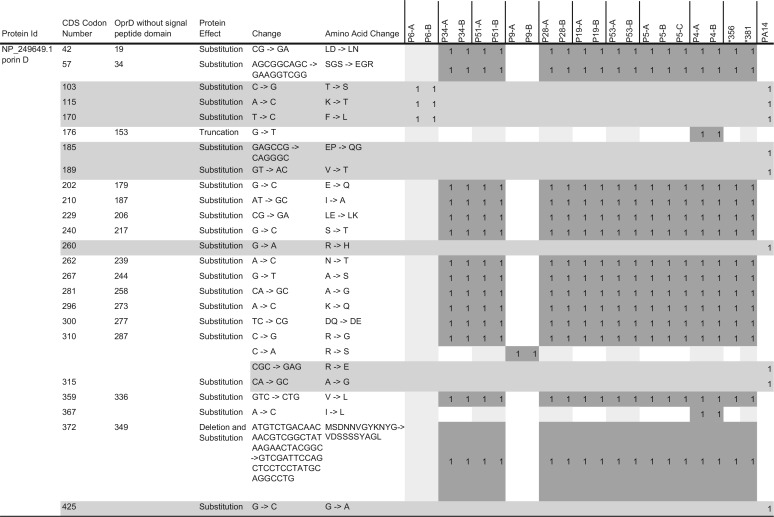
A unique pattern of polymorphic *oprD* genes identified in CF *P. aeruginosa* isolates^*a*^

a The dark-shaded cells represent the mutations that are not shared with PA14. The light-gray-shaded rows represent the polymorphic changes that are shared with PA14 or present in PA14 only.

Table 3 summarizes the *in vitro* MICs of the isolates as well as the nonsynonymous coding changes affecting the number of coding sequences (CDSs) among 9 sets of CF *P. aeruginosa* isolates in reference to the CDSs of PAO1. Intragenic or noncoding sequence mutations were not included in this study. Notably, all 9 of our sets of *P. aeruginosa* genomes were significantly more closely related to their corresponding coisolate(s) than to other *P. aeruginosa* isolates, suggesting that each of them derived from a clonal lineage within the same patient (Fig. 3). The clonal relatedness of isolates appeared to be patient specific regardless of whether they were from the same culture or from temporally different cultures within the 7-month study period (Fig. 3) (Table 4 and 5; see also Table S1 to S4) (a similarly organized whole-genome table is not shown). However, the number of unshared CDS polymorphic changes between the members of coisolate pairs ranged from 302 (P28-A and P28-B) to 536 (P4-A and P4-B), suggesting isogenic differences (Table 3). DK40 isolates of the same clonal type (*356 and *381) from different CF patients showed a significantly higher number (*n* = 10,756) of unshared CDS polymorphic changes between the two. When a value of 5,570 CDSs in PAO1 was used as the reference denominator (excluding all silent or synonymous substitutions and all differences in total CDSs), 2,501 to 2,587 (<50%) nonidentical CDSs were found in the 9 sets of CF *P. aeruginosa*-related lineage pairs (Table 3) (Fig. 3). In contrast, there were 3,838 (69%) nonidentical CDSs in CF *P. aeruginosa* pair *365 and *381 and 4,235 (76%) nonidentical CDSs in all PA14 isolates compared to those of PAO1, which further confirmed that CF *P. aeruginosa* strains *365 and *381 were more closely related to PA14 (Table 3) (Fig. 3). In an analysis in which all nonidentical CDSs were combined from all 9 sets of CF *P. aeruginosa* genomes, a combined total of 4,133 (74%, excluding the ones that were shared with those in PA14) CDSs were found to be different from those in PAO1.

In order to explain heterogeneous MICs between paired isolates, we first examined known antibiotic resistance genes also present in the genomes of PAO1 and PA14. The chromosomal antibiotic resistance genes *ampC*, *ampD*, *ampD*, *ampG*, *dacB*, *bla*_OXA-50_, *aph(3′)-IIb*, and *cat* were compared. There were significant numbers of coding polymorphisms that were specifically shared between clonally related *P. aeruginosa* pairs except for a single unshared point mutation associated with a Y132C substitution in *ampD* of isolate P53-B only and two point mutations with substitutions in *cat* of isolate P5-C only. None of the intra- or interclonal polymorphisms could explain their tested heterogeneous beta-lactam MIC patterns (Table 3; see also Table S2). On the basis of genome coverage, only a 390-kb region of P6-A showed a 3-fold increase in coverage relative to the background genome. This fragment aligned to a 350-kb region of PAO1 chromosome and to 40 kb of the FluMu prophage present in *P. aeruginosa* strain ATCC 27853 (GenBank accession no. CP015117.1), suggesting that the coverage variation was likely due to phage sequence. No other areas of significant coverage variation were observed in the rest of the sequenced isolates.

### Multiple unpaired coding polymorphisms in known quinolone resistance-determining regions (QRDR) were found in clonal pairs.

Highly heterogeneous CIP MICs were observed in coisolate pair P6-A and P6-B from CF patient 6 and coisolate pair P53-A and P53-B from CF patient 53 (Table 4). The classic mutations in the QRDR in *gyrA* codon 83 (T83I and T83A) were detected in both P6-A and P53-B and in an additional 3 isolates (P51-B, P28-A, and P28-B). However, the 3 additional isolates, P51-B, P28-A, and P28-B, did not produce an ultrahigh MIC of >32 μg/ml (Table 4). The lesser-known QRDR mutations in *gyrB* (S466F and S466Y) were also identified in strains P5-C, P4-A, and P4-B, although they showed variable CIP MICs at 1, 1, and 6 μg/ml, respectively, spanning clinical breakpoints of ≤1 μg/ml susceptible (“S”), 2 μg/ml intermediate (“I”), and ≥4 μg/ml resistant (“R”) (Table 4) (31). In fact, none of the amino acid substitutions (absent in PA14) detected in these four genes (*gyrA*, *gyrB*, *parC*, and *parE*) was able to entirely account for either the ultrahigh or ultralow CIP MICs among the 19 isolates measured *in vitro* (Table 4).

### A unique pattern of porin D mutation was found in 15 of the 19 genomes sequenced.

Gene determinants associated with outer membrane porin proteins and efflux pumps and their regulators were highly polymorphic among all isolates (Table 5; see also Table S1 and S2). However, polymorphic mutations in this group of porin and efflux pumps did not indicate the presence of any previously known mechanisms of resistance. For example, despite the highly heterogeneous MICs seen *in vitro* among the 19 isolates, the well-known porin D determinant *oprD* (a total of 443 amino acid residues in PAO1 with a signal peptide of 23 amino acids at the N terminus) was similarly altered among 15 of the 19 isolates (P34-A, P34-B, P51-A, P51-B, P28-A, P28-B, P19-A, P19-B, P53-A, P53-B, P5-A, P5-B, P5-C, P4-A, and P4-B), with strikingly identical patterns even at the nucleotide level (Table 5). Moreover, the same OprD pattern was also shared by two clonally distant reference CF *P. aeruginosa* strains, *356 and *381 (Table 5). All isolates contained identical nonsynonymous substitutions affecting 16 positions, including a well-known outer membrane surface loop 7 variant with a pattern of MSDNNVGYKNYG to VDSSSSYAGL (Table 5) (18).

In addition, an OprD truncation was found in pair P4-A and P4-B due to a stop codon at residue 176 showing nonuniform MIC levels of carbapenems or other agents (Table 3 and 5; see also Table S1). Significantly different OprD coding patterns were associated with two coisolate pairs—P6-A and P6-B shared 3 amino acid substitutions identical to those in PA14, and P9-A and P9-B shared a unique R310S substitution, regardless of the detected intraclonal heterogeneous MICs of both imipenem and meropenem and of other 9 agents tested (Table 3 and 5).

P6-A and P6-B showed ultrahigh SMX-TMP MICs of >32 μg/ml that could be correlated to possible *mexAB-oprM* overexpression resulting from frameshift deletions in negative regulator gene *nalD* at codon 85 (−TGCGCTCGCTCTAC) and at codon 133 (−TG), respectively (32). However, their heterogeneous antimicrobial MICs for all beta-lactam agents (aztreonam, ceftazidime, cefepime, piperacillin-tazobactam, imipenem, and meropenem) could not be simply delineated by the presence of their presumably intact *oprD* gene without considering many additional mutations in other relevant genes and regulators (Table 5; see also Table S1 to S4). In fact, multiple polymorphic mutations in efflux genes (e.g., *mexAB-oprM*, *mexCD-oprJ*, *mexEF-oprN*, *mexXY-oprA*, and *mexJK*) and multiple regulators (e.g., *mexR*, *mexZ*, *nalC*, and *nalD*) were detected in all 19 isolates that were both shared and not shared between intra- and interclonal isolates (Table S1).

### Polymorphic mutations in CDSs associated with *P. aeruginosa* hypermutability and other cellular functions.

Mutations that resulted in high numbers of polymorphic mutations were common not only in isolates containing lesions in DNA mismatch repair genes (*mutL* and *mutS*) but also in those containing lesions in many other determinants. The affected CDSs, including genes involved in nucleotide synthesis (*mutM* and *mutY*), DNA repair or DNA/RNA folding, and recombination functions (*recB*, *recC*, *recD*, *recF*, *recG*, *recJ*, *recN*, *recO*, *rdgC*, *rarA*, *recX*, *recR*, *uvrA*, *uvrB*, *uvrC*, and *uvrD*), were detected in our CF *P. aeruginosa* isolates and in CF *P. aeruginosa* isolates *356 and *381 (Table S3). A 13-bp deletion in *mutS* (Table S3) was found only in P53-B, but this isolate did not have an increased number of significant mutations in CDSs compared to its coisolate P53-A or compared to the rest of 17 *P. aeruginosa* strains (Table 3).

A number of other bacterial cellular functions and known *P. aeruginosa* pathogenicity factors also demonstrated coding variants that were found only in the 19 CF *P. aeruginosa* isolate sequences and not in PAO1 and PA14 (Table 4 to 5; see also Table S1 to S4) (Fig. 3). The affected CDSs included those in genes related to metabolic functions, ABC transporter proteins, electron transport chain functions for ATP synthesis, iron acquisition, two-component sensors and regulators, the RND proteins and membrane porin proteins, transcription regulators, type III secretion systems, DNA repair, RNA polymerization, and structures of flagella and fimbriae, as well as biosynthesis of alginate, pyoverdine, sugar, lipids, nucleotides, amino acids, and vitamins.

## DISCUSSION

Our study demonstrated that antimicrobial MIC heterogeneity is more characteristic of CF *P. aeruginosa* than of non-CF* P. aeruginosa*. We found that both the average number of *P. aeruginosa* coisolates per culture and the average number of agents with discordant susceptibilities between coisolates were higher in the CF cohort than in the non-CF cohort. CF cohort *P. aeruginosa* isolates presented antimicrobial MICs that were more heterogeneous than those seen with non-CF isolates, in the overall analyses as well as in the within-patient and between-patient analyses.

The characteristic phenomenon of the CF *P. aeruginosa* heterogeneous antimicrobial MIC distribution in clonally related isolates is strongly supported by the genomic evidence of a high degree of core genome relatedness as well as unshared coding changes—an average of 412 coding changes—between patient-specific *P. aeruginosa* pairs (Table 3 to 5) (Fig. 2 and 3). We believe that their heterogeneous MICs are the net results of the combined impact of their diverse structural and functional mutations in response to CF host selection. Functional genomic features specific to CF *P. aeruginosa* may not be immediately apparent based on the proportion of CDSs that deviated from those of either PAO1 or PA14, but the unique patterns in specific gene determinants common among CF *P. aeruginosa* isolates may be more informative (Table 3 to 5; see also Table S1 to S4). For example, the mutations that were shared or not shared between the same clonal lineages affecting the known QRDRs had no clear association with CIP MICs (Table 4). In contrast, the unique amino acid substitution patterns, including “loop 7” in OprD, in the majority of CF *P. aeruginosa* isolates could not be mistaken for random events (Table 5) (18). The heterogeneous CF *P. aeruginosa* antimicrobial MICs, especially among clonal pairs, may just be one aspect of the overall adaptive evolution to the host airways (Fig. 1 to 3) (Table 1 to 5; see also Table S1 to S4).

Despite coming from different patients and different lineages, the CF *P. aeruginosa* genomes studied here had similar mutational patterns affecting the same genes (e.g., *oprD*, *mexAB*, *mutL*, and *mutS*), suggesting a common selective pressure (Table 3 to 5; see also Table S3 and S4) (Fig. 3). The unshared changes detected between CF *P. aeruginosa* clonal pairs that occurred in response to host selective pressure are indicative of a transition to a potentially multicellular lifestyle with functional syntrophic associates that are heterogeneous and obligate for growth and resistance (Table 3) (Fig. 1) (3). The plethora of coding polymorphisms (Table 3 to 5; see also Table S1 to S4) detected in CF *P. aeruginosa* isolates is not only responsible for their heterogeneous MIC patterns, but also for a series of adaptive evolutionary process indicative of niche specialization (Fig. 1) (3, 9). The mutations described here will require further characterization *in vitro* and *in vivo* to test these hypotheses.

The overall lack of horizontal gene transfer in CF *P. aeruginosa* is shown by the absence of evidence of areas of sequences with increased coverage (except for P6-A), consistent with the lack of plasmids or other accessory genes that usually drive the emergence of bacterial “superbugs” in free-living bacteria (33, 34). The 19 representative CF *P. aeruginosa* strains all contained a chromosomal *ampC* gene, which, upon induction, is associated with the ability to hydrolyze cephalosporins but not carbapenems, and *bla*_OXA-50_, encoding a constitutively expressed oxacillinase (35). The *aph(3′)-IIb* gene is known to confer resistance to kanamycin and neomycin but not to amikacin, gentamicin, or tobramycin (36). In fact, none of the chromosomal resistance gene determinants [*ampC*, *bla*_OXA-50_, and *aph(3′)-IIb*] or putative regulator mutations in *ampR*, *ampD*, *ampG*, *or dacB* can be used to explain the phenomenon of highly heterogeneous MICs seen with CF *P. aeruginosa* pairs for any single beta-lactam agent tested (Table 3 to 5; see also Table S3). Similar to the 47 *P. aeruginosa* isolates reported by Lee et al. that produced CIP MIC levels of 2 to 64 µg/ml with only the *gyrA* T83I as the “first step” mutation(s) (31), the 19 CF *P. aeruginosa* isolates containing either the well-defined QRDR mutations or novel amino acid substitutions did not show a consistent correlation with their wide range of CIP MICs (Table 4). Quinolone resistance in *P. aeruginosa* has been well characterized as involving porin and efflux mechanisms as well (37, 38). The highly distinct OprD mutational patterns that were conserved within the majority of unrelated CF *P. aeruginosa* isolates (Table 5), along with those associated with the highly polymorphic RND family of efflux proteins and regulators, showed no correlation to previously reported drug transporter or efflux pump activities uniformly affecting *P. aeruginosa* susceptibilities with respect to SMX-TMP, aztreonam, carbapenems, or other classes of antimicrobials (16, 17, 21, 39, 40).

There is ample evidence of the existence of relaxed DNA mismatch repair systems due to mutations in both *mutL* and *mutS* in CF *P. aeruginosa* isolates contributing to “hypermutability” and thus to altered virulence and susceptibilities (9, 13, 29, 41, 42). A previously reported laboratory *P. aeruginosa* strain containing an 11-bp deletion in *mutS* was found to show increases in the rate of base pair mutations of 267× and in the rate of indels of 230× in comparison to a wild-type founder *P. aeruginosa* strain under culture conditions (43). On the basis of the variants seen in the 19 CF *P. aeruginosa* isolates, we believe that the hypermutable state of CF *P. aeruginosa* strains cannot be solely attributed to diverse mutations in *mutL* and/or *mutS*, as a *mutS* deleterious frameshift mutation in an isolate from P53-B did not show increased total numbers of mutations or CDSs affected in comparison to the other 18 isolates (Table 3; see also Table S1 to S4). Our additional findings of mutations in determinants responsible for nucleotide synthesis, DNA/RNA modification, and recombination enzymes further support the long-standing observation of the “genome decay” phenomenon as part of the bacterial symbiotic response to host selective pressure (Table S4) (3, 10, 44).

The current state of clinical laboratory susceptibility reporting has strictly followed the conventions of the three categories of interpretations for *in vitro* antimicrobial activities (“S,” “I,” and “R”). These interpretations have played and will continue to play a significant role in providing clinical reference values and guiding treatment decisions. These categories are accurate for rapidly growing bacterial strains that are phenotypically homogeneous, likely due to their brief encounters with the host organisms in settings of “acute” infections. Although content is limited with respect to heteroresistance, CLSI has also developed technical instructions such as “examine carefully with transmitted light for >1 colony or light film of growth” regarding recording zones of inhibition around oxacillin/cefoxitin against staphylococcus and streptococcus (7). These instructions have begun to address susceptibility characteristics that are beyond the clear-cut “S,” “I,” and “R” categories. However, heterogeneous susceptibilities generated from colony variants of the same culture have not received elevated recognition. Before we can embark on a proper laboratory report with treatment implications, we propose the use of broad-range microdilutions for susceptibility testing of bacterial isolates exhibiting slow and heterogeneous growth patterns *in vitro*. We also propose the use of a proper form of laboratory documentation in the presence of heterogeneous MICs.

This study was limited by the overall small number of isolates used for statistical analysis and the lack of information regarding the duration of *P. aeruginosa* infection in the members of either cohort. Neither patient underlying diseases nor patient treatment history was included in this analysis. The non-CF cohort of *P. aeruginosa* isolates may have included patients whose pseudomonal infections were similar to those of patients with CF or those of patients who have had prolonged infections affecting other organ or tissue systems. Such mixed inclusion would bias results toward the null instead of exacerbating the effects seen here. Future studies separating *P. aeruginosa* isolates collected from hosts with newly acquired infections and isolates collected from hosts with chronic infections (both CF and non-CF) would improve data clarity. Bacterial whole-genome longitudinal analysis of isolates will be able to recapitulate the process by which continuous host-selected genome decay leads to the rise of a “host tissue-definitive” *P. aeruginosa* multicellular specialist and to confirm the lack of repeat wild-type *P. aeruginosa* infections thereafter.

The term “heteroresistance” may represent just one specific segment on the continuum of heterogeneous MICs. The finding of disparate intraclonal antimicrobial MICs *in vitro* may be explained by bacterial functional divergence with increased interdependence of growth and resistance during niche specialization (Fig. 1 and 2) (14). With the advances in treatment of congenital or acquired defects and many other complex diseases, there is a growing patient population receiving frequent and long-term antibiotic therapy that may be at increased risk of developing chronic focal infections (14). The acknowledgment and understanding of laboratory findings of bacterial discordant susceptibilities and/or heteroresistance in the setting of chronic focal infections can open opportunities for the use of alternative therapeutic evaluations and approaches (3).

## MATERIALS AND METHODS

### Bacterial isolates from cultures included for antimicrobial MIC analysis.

This study included all clinically obtained *P. aeruginosa* isolates identified in the Clinical Microbiology Laboratory at Seattle Children’s Hospital (SCH) over a 7-month period (June 2013 to December 2013) and was approved by the SCH Institutional Review Board. All *P. aeruginosa* isolates were identified by matrix-assisted laser desorption ionization–time of flight (MALDI-TOF) analysis (Bruker Daltonics Inc.). Culture methods for isolates from CF patients were distinct from those used with non-CF isolates as previously described (9, 45). Colony variants of all bacterial isolates were separated from mixed cultures using single-colony subcultures based on size, pigment, texture, hemolysis patterns, mucoidy, etc. Potential susceptibilities associated with a nonhomogeneous appearance were reexamined after maximal purification of colony variants for repeat susceptibility testing. Where Etest patterns similar to those shown in Fig. 1A and B were reproducible, “heteroresistance” was noted; otherwise, separate susceptibility data are reported for isolates showing homogeneous growth and susceptibilities.

### Antibiotic susceptibility testing and MIC determination.

Antibiotic susceptibility was determined by the Etest (BioMérieux) MIC method. All isolates were tested for susceptibility to amikacin, gentamicin, tobramycin, aztreonam, ceftazidime, cefepime, imipenem, meropenem, piperacillin-tazobactam, CIP, and SMX-TMP. Each single isolate from mixed cultures was maximally purified by the use of single-colony subcultures based on colony size, hemolysis, pigment, texture, mucoidy, etc. Etest susceptibility testing was set up as previously described (9). In general, the Etest plates were incubated at 35°C in ambient air for 18 to 24 h. Prolonged incubation was frequently required for some CF *P. aeruginosa* isolates due to slow growth (requiring up to 36 to 48 h) for MIC determination. Antimicrobial susceptibility data were interpreted using CLSI M100-S23 MIC breakpoint criteria (46).

### Statistical analyses of variability in antimicrobial MICs.

We first assigned detection limits corresponding to those MIC data points for which the highest or the lowest detection limits were reached (e.g., an MIC value of >256 was entered as a value of 256). We then applied logarithmic transformation to all the MIC readings (logMICs) to make the data less skewed for analysis. All subsequent analyses were based on logMICs.

Since multiple isolates from the same patients were collected and tested in the same period, we applied repeated analysis of variance (ANOVA) to decompose the data corresponding to the total variance in logMICs into the categories of variance within patients (among isolates from the same patient) and variance between patients. The repeated ANOVA was conducted separately for CF and non-CF patients. In order to statistically compare the differences in the levels of logMIC variability between the CF and non-CF patients, we derived 95% bootstrap confidence intervals for the within- and between-patient variations, as well as for the total variations. We decided *a priori* that two variance estimates were significantly different if their corresponding confidence intervals did not overlap. All statistical analyses were performed using Stata version 12 (Stata Corp., College Station, TX).

### Bacterial whole-genome sequencing.

A subset of *P. aeruginosa* isolate pairs was selected for whole-genome sequencing based on their heterogeneous or discordant *in vitro* antimicrobial MICs for at least 1 of the 11 agents tested (Table 4). All 19 CF *P. aeruginosa* isolates were verified both by naked eye and by microscopic appearance on Mueller Hinton (MH) and M9 minimal media to produce relatively homogeneous growth *in vitro*. Isolates showing nonhomogeneous growth patterns (Fig. 1) were not included in the sequencing investigation. The control genomes of strains PAO1 (NC_002516.2) and UCBPP-PA14 (PA14; NC_008463.1) and two previously sequenced genomes of clonally related CF *P. aeruginosa* reference strains *356 and *381 (SRA accession ERS402683 and ERS402710, respectively) from a European study were included for this analysis (10). PAO1 and PA14 are used to represent the two highly dissimilar *P. aeruginosa* genomes for analysis as non-CF *P. aeruginosa* references or “wild-type” susceptibility controls (9, 13, 47). All sequence polymorphisms associated with our CF *P. aeruginosa* strains (i.e., strains *356 and *381) as well as with PA14 were generated in reference to the PAO1 genome (10). In this study, polymorphisms found in PA14 in reference to PAO1 were assumed to have minimal functional impact on structural or biological functions, including *in vitro* susceptibilities, if both were treated as non-CF “wild-type” strains (Table 4 and 5; see also Table S1 to S4).

DNA was extracted from *P. aeruginosa* isolates and diluted to 1 ng/μl for sequencing library generation with third-volume Nextera XT and 14 cycles of PCR amplification (48, 49). Sequencing reads were adapter and quality trimmed using cutadapt, *de novo* assembled using SPAdes v3.9, and annotated using prokka (50, 51). Reads were mapped to the PAO1 reference genome (NC_002516.2), and variant call files were generated using Geneious v9.1 and cutoff values of at least 7× coverage and 70% allele frequency. Core genome trees were generated based on single nucleotide variants that excluded indels as described previously (52).

### Accession number(s).

Bacterial assemblies were deposited in NCBI GenBank under BioProject PRJNA369567.
